# Association between continuous metabolic syndrome score and incident dementia: a longitudinal study of middle-aged and older Chinese adults

**DOI:** 10.1186/s12883-026-05054-6

**Published:** 2026-07-03

**Authors:** Ling Lin, Hua Liu, Dan Long, Chaofeng Fan

**Affiliations:** Department of Neurosurgery, West China Hospital, Sichuan University, No.37 Guoxue Lane, Wuhou District, Chengdu, 610041 China

**Keywords:** Metabolic syndrome, Dementia, CHARLS, Longitudinal study, Aging

## Abstract

**Background:**

While metabolic syndrome (MetS) is a well-established driver of cognitive decline, conventional dichotomous diagnostic criteria fail to capture the cumulative and synergistic allostatic load of metabolic dysfunction. We investigated the longitudinal association between an age- and sex-standardized continuous MetS severity score and risk of incident dementia in a nationally Chinese cohort.

**Methods:**

This prospective analysis utilized data from the China Health and Retirement Longitudinal Study. A continuous MetS score was calculated at baseline integrating waist circumference, triglycerides, high-density lipoprotein cholesterol, mean arterial pressure, and fasting blood glucose via validated linear models, and then quartiled (lowest quartile as reference). Incident dementia was ascertained during the Wave 4 follow-up using a rigorous, multidimensional diagnostic protocol. The dose–response relationship was evaluated using restricted cubic splines (RCS) and logistic regression.

**Results:**

Among 4,920 dementia-free participants at baseline, 667 (13.56%) developed dementia over the follow-up. RCS modeling revealed a significant, linear dose–response association between increasing MetS scores and dementia risk. Each 1-SD increment in the MetS score was independently associated with a 12% higher risk of dementia (OR = 1.12, 95% CI: 1.01–1.24). Participants in the highest quartile group faced a 48% increased risk compared to those in the lowest quartile (OR = 1.48, 95% CI: 1.07–2.04). This risk amplification was uniquely pronounced among non-obese individuals (BMI < 24 kg/m^2^; P-interaction = 0.378).

**Conclusions:**

An elevated continuous MetS score is a robust, independent predictor of incident dementia in middle-aged and older adults. Transitioning from binary criteria to a continuous MetS metric provides a nuanced, dose-dependent assessment of dementia risk.

**Supplementary Information:**

The online version contains supplementary material available at https://doi.org/10.1186/s12883-026-05054-6.

## Background

As the global population ages, dementia has emerged as a paramount public health challenge [1]. While recent pharmacological advancements have introduced therapies capable of slowing Alzheimer's disease progression, definitive treatments that can fully halt or reverse neurodegeneration remain elusive. Consequently, delaying the onset of neurodegeneration through the aggressive management of modifiable cardiometabolic risk factors during mid-to-late life remains the cornerstone of preventive neurogeriatrics [2].

Metabolic syndrome (MetS)—a multifaceted clustering of central adiposity, dyslipidemia, hypertension, and impaired glucose tolerance—is a recognized determinant of structural brain aging and cognitive impairment [3]. However, current epidemiological frameworks in neurocognitive research predominantly rely on traditional, dichotomous diagnostic criteria for MetS (e.g., those from the NCEP-ATP III or the International Diabetes Federation) [4–7]. Recent neuro-epidemiological literature highlights these threshold-based definitions have inherent methodological limitations: they treat metabolic health as a binary state, assign equal pathological weight to each component, and often overlook individuals with subclinical cerebrovascular deterioration who fall just below the diagnostic threshold [8, 9].

In the context of dementia research, studies using binary MetS criteria have yielded inconsistent findings, with some reporting null or weak associations with cognitive decline and incident dementia [6, 7]. This heterogeneity likely stems from the loss of dose–response information and the misclassification of individuals with partial metabolic dysfunction as “metabolically healthy”. Recent advancements in metabolic epidemiology have addressed these constraints through the development of a continuous MetS severity score [10, 11]. Validated rigorously within Chinese populations, this metric utilizes tailored factor loadings to quantify the precise continuum of metabolic dysregulation [9]. While its predictive superiority for cardiovascular events is established, its prognostic utility for neurodegenerative outcomes requires comprehensive investigation.

Therefore, the present study aimed to elucidate the longitudinal association between this continuous MetS score and incident dementia utilizing a nationally representative cohort of middle-aged and older Chinese adults. Furthermore, we sought to explore the modifying effects of age, sex, obesity, and lifestyle behaviors on this association.

## Methods

### Study design and participants

Data were extracted from the China Health and Retirement Longitudinal Study (CHARLS), an ongoing, nationally representative prospective cohort tracking the health trajectories of community-dwelling Chinese adults aged 45 years and older [12]. The baseline survey (Wave 1, 2011) employed a multistage, stratified probability-proportional-to-size sampling design spanning 150 counties across 28 provinces. The study protocol was approved by the Biomedical Ethics Review Committee of Peking University (IRB00001052-11015) in accordance with the Declaration of Helsinki, with written informed consent secured from all participants [13].

**Fig. 1:**
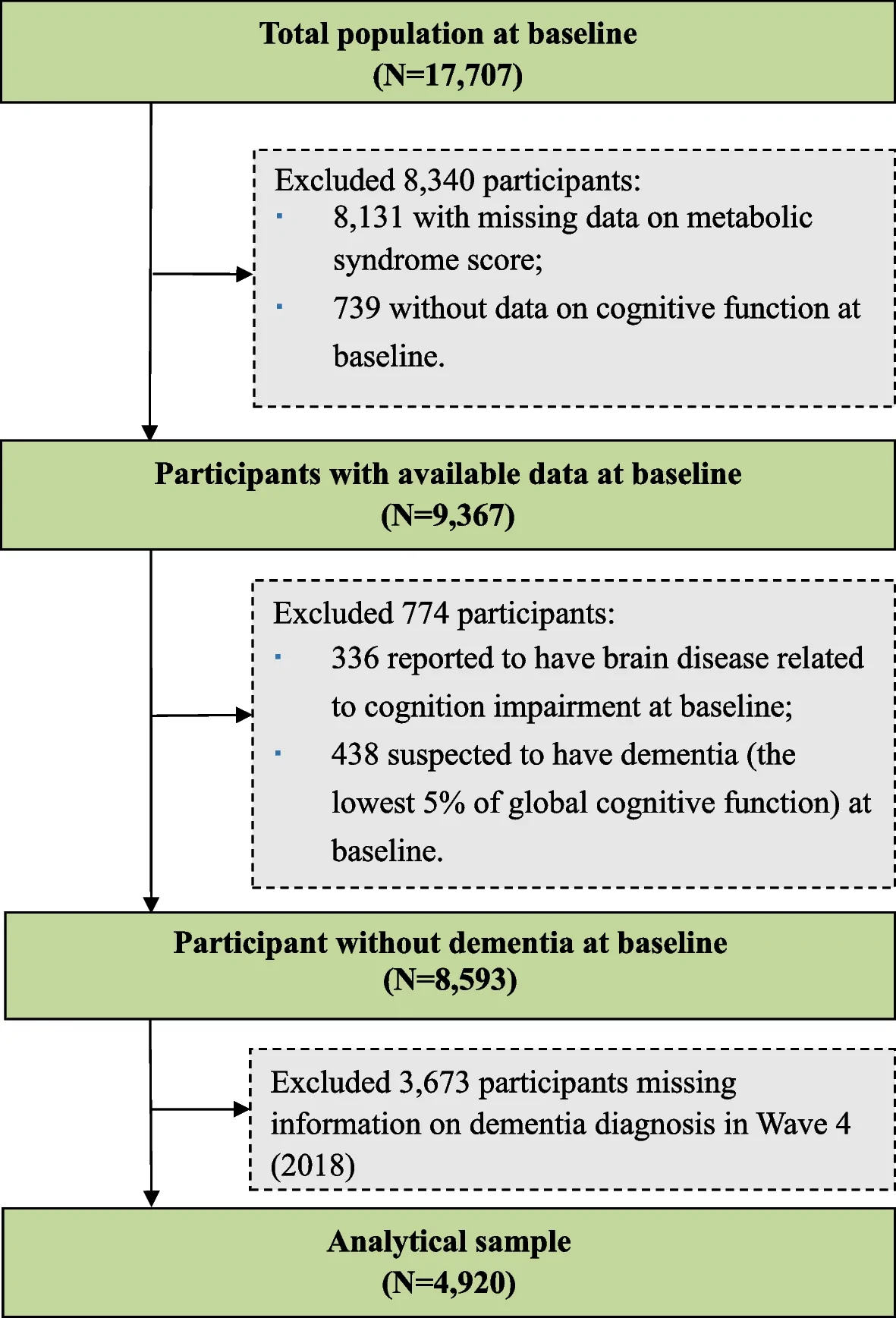
Flowchart of the study population

From the initial 17,707 enrollees, we established a rigorous, dementia-free analytical cohort. Participants were excluded for missing data on critical metabolic parameters or baseline cognition (*n* = 8,340). To mitigate reverse causality, individuals with pre-existing brain diseases linked to cognitive impairment or suspected baseline dementia (lowest 5% of global cognitive scores) were excluded (*n* = 774). Following the exclusion of participants lacking dementia ascertainment in the 2018 follow-up (Wave 4), the final longitudinal cohort comprised 4,920 individuals. The detailed selection process is illustrated in Fig. 1.

### Data collection

Standardized face-to-face interviews captured sociodemographic characteristics, lifestyle behaviors, and health status [13]. Meanwhile, professional medical personnel gathered anthropometric data (height, weight, and waist circumference [WC]) and blood samples in accordance with established protocols.

Demographic variables included age, sex, place of residence (urban or rural), and marital status (grouped as married/cohabitating or other). Educational attainment was divided into three tiers: no formal education, middle school and below, and high school and above. Body mass index (BMI) was calculated as weight divided by height squared (kg/m^2^), and is categorized as underweight (< 18.5), normal weight (18.5–23.9), overweight (24.0–27.9), and obesity (≥ 28.0). Assessment of lifestyle behaviors encompassed smoking status (never, former, or current), alcohol consumption (drinker or non-drinker), and social activity (categorized as active or inactive based on participation during the month before the interview) [14]. We assigned a point of 1 to each unhealthy habit—specifically current or former smoking, alcohol consumption, and social inactivity—and 0 to healthy ones. The aggregate lifestyle score, ranging from 0 to 3, was calculated by summing these points. For stratified analysis, participants were split into a favorable lifestyle group (score 0–1) and an unfavorable lifestyle group (score 2–3).

Continuous clinical measurements, including systolic and diastolic blood pressure, fasting blood glucose (FBG), triglycerides (TG), and high-density lipoprotein cholesterol (HDL-C), were extracted from baseline physical examinations and venous blood sample analyses. Prevalent chronic conditions (hypertension, diabetes, cardiovascular disease, and dyslipidemia) were systematically identified via clinical measurements, concurrent medication usage, and self-reported physician diagnoses. Hypertension was defined by a history of the condition, the use of antihypertensive drugs, or measured blood pressure ≥ 140/90 mmHg. Diabetes was determined through diagnostic history, use of glucose-lowering medication, fasting blood glucose ≥ 126 mg/dL, or HbA1c ≥ 6.5%. Cardiovascular disease (CVD) was defined as a history of stroke or heart disease (including heart attack, angina, or heart failure). Dyslipidemia was identified via self-reported diagnosis, treatment, or abnormal lipid profiles (total cholesterol ≥ 200 mg/dL or TG ≥ 150 mg/dL).

Baseline global cognition was quantified using a validated instrument encompassing orientation, episodic memory, and executive function. The global cognition score was computed as the sum of these domain scores (range 0–21), with higher values indicating superior cognitive performance [15].

### Assessment of MetS score

Metabolic severity was operationalized via a continuous MetS score, utilizing an established methodology specific to the Chinese population [9]. The calculation integrated five distinct physiological parameters, notably including waist circumference (WC) as a primary metric of central adiposity, alongside TG, HDL-C, MAP (mean arterial pressure), and FBG. Age- and sex-specific linear scoring formulas, derived from weighted factor analysis loadings, were applied to compute a normalized continuum of metabolic dysfunction. The score was derived using age- and sex-stratified equations that apply specific weighting coefficients to each metabolic parameter. For example, mean arterial pressure (MAP) was calculated as DBP + (SBP—DBP)/3. The exact linear scoring formulas for each demographic subgroup (e.g., For males aged < 60 years: MetS score = −2.9092 + 0.0262*WC + 0.3098*TG—0.9440*HDL-C + 0.0097*MAP + 0.0745*FBG) are comprehensively detailed in the Supplementary Methods. The internal and external validity of these scoring models have been rigorously confirmed using the China Multi-Ethnic Cohort (CMEC) and three independent provincial datasets (*N* = 134,403), demonstrating high predictive accuracy for future cardiovascular disease risk across different population subgroups.

### Assessment of dementia

Incident dementia was assessed during the Wave 4 follow-up (2018) employing the Harmonized Cognitive Assessment Protocol (HCAP), analogous to the English Longitudinal Study of Ageing (ELSA) criteria —an approach with established acceptable sensitivity and specificity (sensitivity 0.58, specificity 0.98) for population-based screening [16–18]. This multidimensional algorithm synthesized three diagnostic pillars: (1) objective cognitive impairment (scoring < 1.5 standard deviations below age- and education-specific normative means in ≥ 2 domains; orientation, episodic memory, executive function [interpreted logical reasoning], and verbal fluency [animal-naming task]); (2) informant-reported cognitive decline (Informant Questionnaire on Cognitive Decline in the Elderly [IQCODE] score ≥ 3.6); and (3) functional disability (impairment in Activities of Daily Living [ADL]). Probable dementia was adjudicated if a participant exhibited objective cognitive impairment or a clinically significant IQCODE score, coupled with concurrent ADL impairment, or if they had a corroborated clinical diagnosis of Alzheimer’s disease.

### Statistical analysis

Baseline characteristics were stratified by MetS score quartiles (Q1–Q4) and compared using analysis of variance (ANOVA) for continuous variables and chi-square tests for categorical variables. The dose–response association between the continuous MetS score and incident dementia was modeled using restricted cubic splines (RCS).

Multivariable logistic regression was utilized to compute odds ratios (ORs) and 95% confidence intervals (CIs). MetS was analyzed both as a continuous variable per 1-SD increment and categorized into quartiles. Model 1 was adjusted for fundamental demographic factors (age, sex, and education). Model 2 incorporated extensive adjustments for marital status, residence, BMI, smoking status, alcohol drinking, social activity, hypertension, diabetes, cardiovascular disease, and dyslipidemia. To test for linear trends across categories, the ordinal variable representing the MetS score quartiles (coded 1 through 4) was entered into the multivariable logistic regression models as a continuous variable, and the resulting *P*-value was reported as the P for trend. Subgroup analyses were performed across age (< 60 vs. ≥ 60), sex, BMI (< 24vs. ≥ 24), and lifestyle strata (favorable vs. unfavorable), with statistical interactions assessed via cross-product terms. Unweighted regression models were utilized as the primary objective of this study was analytical (assessing the biological association between MetS and dementia) rather than descriptive. Furthermore, adjustment for demographic variables linked to the sampling design (e.g., age, sex, residence) was deemed sufficient to minimize confounding while preserving statistical efficiency in this restricted sub-cohort.

Sensitivity analyses were carried out to verify the robustness of the primary findings. First, we additionally controlled for the baseline cognition score to rule out reverse causality. Second, to address potential bias caused by missing data, multiple imputation by chained equations (MICE) was performed to generate 10 imputed datasets, and the results were pooled according to Rubin’s rules. A two-sided *P*-value of < 0.05 was considered statistically significant. All statistical analyses were performed using Stata SE 16.0 (Stata Corp, College Station, Texas).

## Results

### Characteristics of the study population

The analytical cohort consisted of 4,920 participants (mean age 62.11 ± 6.89 years). Baseline characteristics across the MetS score quartiles are delineated in Table 1. While age, marital status, and baseline cognitive scores were statistically uniform across the distribution, striking demographic and clinical gradients were observed. Participants in the highest MetS quartile (Q4) were predominantly female and urban-dwelling. Concurrently, Q4 participants exhibited dramatically higher prevalences of generalized obesity, hypertension, diabetes, cardiovascular disease, and dyslipidemia.

**Table 1:** Characteristics of the study population by quartiles of metabolic syndrome (MetS) score (*N* = 4,920)

Characteristics	Q1 (Lowest)	Q2	Q3	Q4 (Highest)	P
N	1,266	1,167	1,245	1,242	-
Age, years	61.99 ± 7.18	62.14 ± 7.06	62.22 ± 6.64	62.09 ± 6.67	0.870
Female, n (%)	435 (34.36)	571 (48.93)	676 (54.30)	824 (66.34)	< 0.001
Education, n (%)					0.047
No formal education	340 (26.88)	367 (31.45)	373 (29.96)	407 (32.77)	
Middle school	618 (48.85)	521 (44.64)	567 (45.54)	560 (45.09)	
High school and above	307 (24.27)	279 (23.91)	305 (24.50)	275 (22.14)	
Married, n (%)	1113 (87.91)	1020 (87.40)	1090 (87.55)	1097 (88.33)	0.900
Rural residence, n (%)	962 (75.99)	830 (71.12)	783 (62.89)	714 (57.49)	< 0.001
BMI, kg/m^2^	20.88 ± 2.73	22.51 ± 3.14	24.22 ± 3.76	25.99 ± 3.67	< 0.001
Underweight	203 (16.18)	76 (6.57)	36 (2.90)	13 (1.06)	
Normal weight	926 (73.78)	765 (66.12)	595 (47.98)	346 (28.15)	
Overweight	114 (9.08)	279 (24.11)	454 (36.61)	540 (43.94)	
Obesity	12 (0.96)	37 (3.20)	155 (12.50)	330 (26.85)	
Smoking, n (%)					< 0.001
Never	583 (46.05)	684 (58.61)	761 (61.12)	864 (69.57)	
Former	111 (8.77)	107 (9.17)	128 (10.28)	119 (9.58)	
Current	572 (45.18)	376 (32.22)	356 (28.59)	259 (20.85)	
Alcohol drinking, n (%)					< 0.001
Never	664 (52.45)	786 (67.35)	888 (71.33)	953 (76.73)	
Drinker	602 (47.55)	381 (32.65)	357 (28.67)	289 (23.27)	
Social activity, n (%)					< 0.001
Inactive	722 (57.03)	605 (51.84)	656 (52.69)	574 (46.25)	
Active	544 (42.97)	562 (48.16)	589 (47.31)	667 (53.75)	
Hypertension, n (%)	346 (27.33)	402 (34.45)	613 (49.24)	776 (62.48)	< 0.001
Diabetes, n (%)	98 (7.74)	125 (10.71)	194 (15.58)	437 (35.19)	< 0.001
Cardiovascular disease, n (%)	97 (8.82)	125 (12.35)	130 (12.33)	202 (19.27)	< 0.001
Dyslipidemia, n (%)	541 (43.18)	466 (40.35)	583 (47.36)	694 (56.70)	< 0.001
MetS Score	−0.80 ± 0.37	−0.08 ± 0.16	0.46 ± 0.18	1.55 ± 0.94	< 0.001
Cognition score	10.61 ± 3.72	10.85 ± 3.83	10.98 ± 3.87	10.92 ± 3.78	0.078

Values are mean ± standard deviation or n (%)

*Abbreviations*: *BMI* body mass index

### Association between MetS score and dementia

**Fig. 2:**
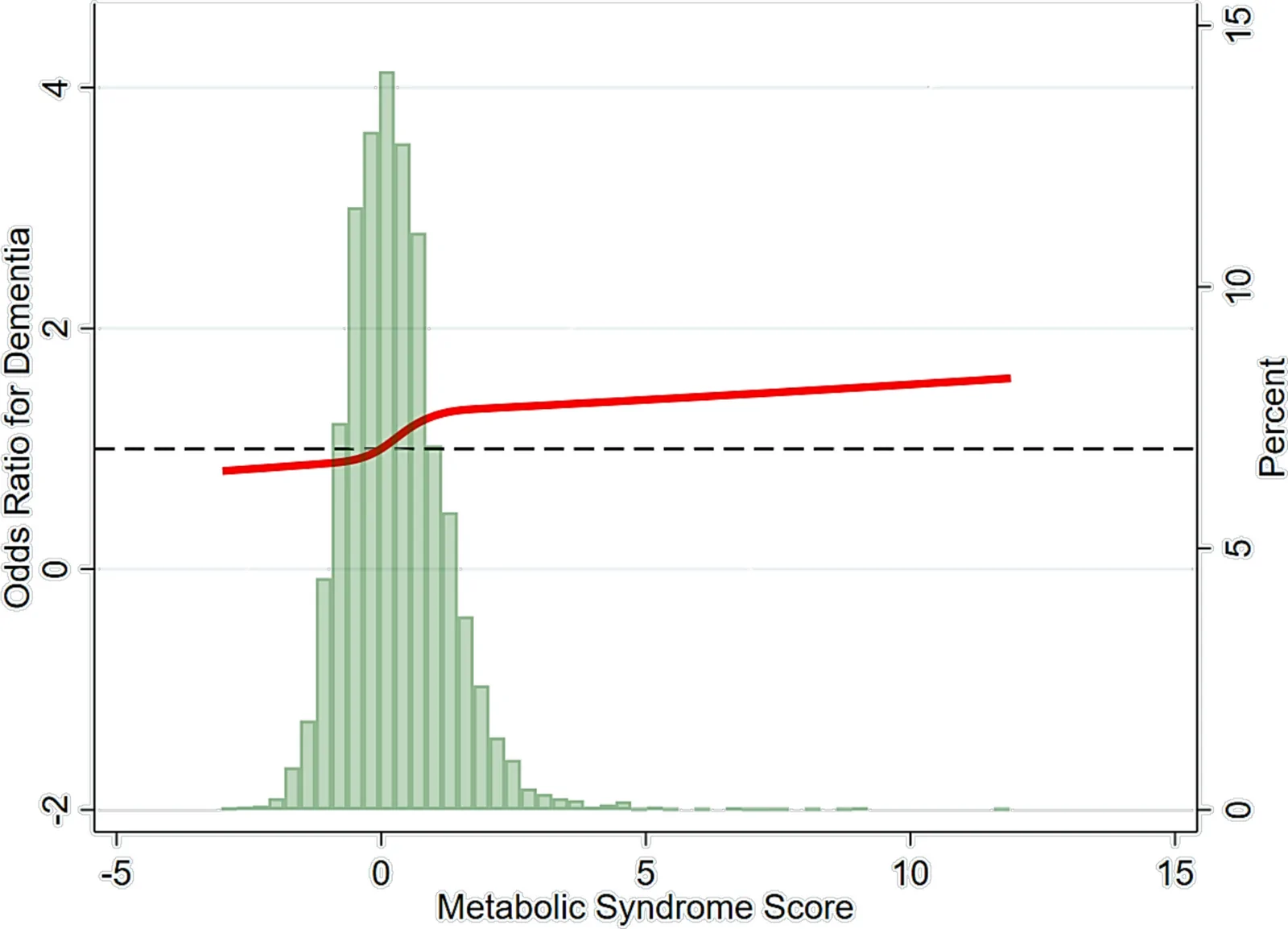
Dose–response relationship between metabolic syndrome (MetS) score and dementia risk: a restricted cubic spline analysis

Over the follow-up period, incident dementia was identified in 667 participants (13.56%), with the incidence rate increasing consistently from the lowest quartile (10.98% in Q1) to the highest quartile (16.43% in Q4). RCS modeling elucidated a significant, positive linear dose–response association linking the continuous MetS score to an escalated risk of dementia (*P for non-linearity* = 0.386; Fig. 2).

Further multivariable logistic regression analyses confirmed this significant association (Table 2). After adjusting for an exhaustive array of demographic, behavioral, and clinical confounders (Model 2), each 1-SD increment in the MetS score independently conferred a 12% higher risk of incident dementia (OR = 1.12, 95% CI: 1.01–1.24). Categorical analysis revealed a progressive risk continuum: relative to the lowest quartile (Q1), individuals situated in the highest MetS quartile (Q4) experienced a 48% higher risk of dementia (OR = 1.48, 95% CI: 1.07–2.04), with a significant linear trend (*P* = 0.017).

**Table 2:** Odds ratios (ORs) and 95% confidence intervals (CIs) for the association between metabolic syndrome (MetS) score and subsequent dementia

**MetS Score**	**No. of participants**	**No. of cases**	**Incidence rate (%)**	**Model 1**^a^		**Model 2**^b^	
				**OR (95% CI)**	***P***	**OR (95% CI)**	***P***
Continuous	4,920	667	13.56	1.17 (1.08, 1.26)	< 0.001	1.12 (1.01, 1.24)	0.035
Categorical							
Q1 (Lowest)	1,266	139	10.98	1.00 (Reference)	-	1.00 (Reference)	-
Q2	1,167	153	13.11	1.21 (0.94, 1.56)	0.137	1.08 (0.81, 1.45)	0.602
Q3	1,245	171	13.73	1.29 (1.01, 1.65)	0.041	1.17 (0.87, 1.58)	0.300
Q4 (Highest)	1,242	204	16.43	1.60 (1.26, 2.04)	< 0.001	1.48 (1.07, 2.04)	0.018
P for trend					< 0.001		0.017

^a^Adjusted for age, sex and education

^b^Adjusted for age, sex, education, marital status, residence, body mass index, smoking, alcohol drinking, social activity, hypertension, diabetes, cardiovascular disease, and dyslipidemia

### Stratified analyses

The deleterious impact of an elevated MetS score on dementia risk remained directionally consistent across all evaluated demographic and lifestyle subgroups (Table 3), yielding no statistically significant multiplicative interactions (all *P for interaction* > 0.05).

**Table 3:** Odds ratios (ORs) and 95% confidence intervals (CIs) for the association between metabolic syndrome (MetS) score and subsequent dementia: stratified by age, sex, BMI, and lifestyle

MetS Score	No. of participants	No. of cases	OR (95% CI)	*P-value*	*P for interaction*
*Age* < *60*					0.064
Continuous	2,102	174	1.21 (1.03, 1.42)	0.020	
Q1 (Lowest)	580	35	1.00 (Reference)	-	
Q2	514	37	0.98 (0.56, 1.70)	0.929	
Q3	504	37	0.96 (0.53, 1.73)	0.883	
Q4 (Highest)	504	65	1.67 (0.90, 3.10)	0.107	
*Age* ≥ *60*					
Continuous	2,818	493	1.05 (0.92, 1.20)	0.487	
Q1 (Lowest)	686	104	1.00 (Reference)	-	
Q2	653	116	1.16 (0.82, 1.64)	0.398	
Q3	741	134	1.25 (0.88, 1.77)	0.213	
Q4 (Highest)	738	139	1.33 (0.91, 1.95)	0.412	
*Female*					0.592
Continuous	2,506	364	1.08 (0.95, 1.22)	0.259	
Q1 (Lowest)	435	50	1.00 (Reference)	-	
Q2	571	83	1.30 (0.85, 1.99)	0.227	
Q3	676	93	1.37 (0.89, 2.09)	0.152	
Q4 (Highest)	824	138	1.63 (1.05, 2.54)	0.030	
*Male*					
Continuous	2,414	303	1.28 (1.05, 1.56)	0.014	
Q1 (Lowest)	831	89	1.00 (Reference)	-	
Q2	596	70	0.89 (0.59, 1.36)	0.595	
Q3	569	78	1.06 (0.68, 1.64)	0.812	
Q4 (Highest)	418	66	1.49 (0.90, 2.49)	0.123	
*BMI* < *24 kg/m*^*2*^					0.378
Continuous	2,960	394	1.23 (1.07, 1.41)	0.004	
Q1 (Lowest)	1,129	123	1.00 (Reference)	-	
Q2	841	109	1.06 (0.76, 1.47)	0.732	
Q3	631	94	1.25 (0.89, 1.77)	0.200	
Q4 (Highest)	359	68	1.86 (1.25, 2.79)	0.002	
*BMI* ≥ *24 kg/m*^*2*^					
Continuous	1,921	265	1.02 (0.87, 1.20)	0.803	
Q1 (Lowest)	126	14	1.00 (Reference)	-	
Q2	316	42	0.99 (0.48, 2.08)	0.997	
Q3	609	77	0.89 (0.45, 1.78)	0.746	
Q4 (Highest)	870	132	1.09 (0.55, 2.14)	0.811	
*Favorable lifestyle*					0.603
Continuous	3,068	412	1.12 (0.99, 1.27)	0.057	
Q1 (Lowest)	606	68	1.00 (Reference)	-	
Q2	728	93	1.13 (0.77, 1.67)	0.533	
Q3	821	102	1.14 (0.77, 1.70)	0.510	
Q4 (Highest)	913	149	1.61 (1.07, 2.41)	0.023	
*Unfavorable lifestyle*					
Continuous	1,851	255	1.11 (0.91, 1.36)	0.313	
Q1 (Lowest)	660	71	1.00 (Reference)	-	
Q2	439	60	1.02 (0.65, 1.61)	0.934	
Q3	424	69	1.28 (0.81, 2.03)	0.296	
Q4 (Highest)	328	55	1.20 (0.69, 2.07)	0.517	

Adjusted for age, sex, education, marital status, residence, body mass index, smoking, alcohol drinking, social activity, hypertension, diabetes, cardiovascular disease, and dyslipidemia if applicable

However, exploratory analyses focusing on BMI strata revealed a clinically profound divergence in point estimates. The higher risk of dementia in relation to MetS (Q4) was exceptionally pronounced among non-obese participants (BMI < 24kg/m^2^; OR = 1.86, 95% CI: 1.25–2.79) than overweight/obese participants (BMI ≥ 24 kg/m^2^; OR = 1.09, 95% CI: 0.55–2.14). However, given the non-significant interaction terms, these numerical differences should be interpreted with caution.

### Sensitivity analysis

The results remained consistent after using multiple imputation for missing covariates (Supplementary Table 1) and after further adjustment for baseline cognition scores (Supplementary Table 2).

## Discussion

In this large-scale, prospective analysis of middle-aged and older Chinese adults, we demonstrated that an escalating continuous metabolic syndrome score is a potent, independent predictor of incident dementia. By modeling metabolic severity as a linear continuum rather than a binary threshold, we observed that individuals in the highest quartile of allostatic load faced a 48% higher risk of dementia. Crucially, these associations remained robust after comprehensive adjustment for baseline cognitive status, sociodemographic factors, and established cardiometabolic diseases.

Our findings offer a distinct advantage over prior epidemiological research utilizing dichotomous definitions. For instance, previous studies in Chinese cohorts applying binary NCEP-ATP III criteria have occasionally yielded equivocal or statistically attenuated associations with cognitive decline, largely because threshold-based definitions assign equal pathological weight to each component and classify sub-clinically impaired individuals as 'healthy' [8]. By transitioning to a continuous metric, our study minimizes this phenotypic misclassification, capturing the synergistic toxicity of clustered metabolic abnormalities and demonstrating a highly significant, linear dose–response relationship with incident dementia. Recent neuroimaging studies complement our clinical findings, demonstrating that continuous metrics of metabolic severity correlate precisely with accelerated brain atrophy, diminished hippocampal volume, the proliferation of white matter hyperintensities, and cerebral haemodynamics [3, 19]. By deploying an age- and sex-standardized continuous score, our study minimizes phenotypic misclassification and definitively maps the trajectory of metabolic deterioration to neurocognitive vulnerability.

While the continuous MetS score effectively evaluates cumulative allostatic load, its clinical translation requires examining its individual weighted components. The factor loadings in our underlying scoring equations demonstrate that dyslipidemia (specifically elevated TG and reduced HDL-C) and WC exert disproportionately large statistical weights compared to mild elevations in fasting glucose. Clinically, this suggests that in middle-aged and older adults exhibiting high continuous MetS scores, early and aggressive lipid management and the reduction of visceral adiposity should be prioritized to mitigate subsequent cerebrovascular risk and neurodegeneration.

The mechanistic underpinnings linking systemic metabolic severity to neurodegeneration are complex and interrelated. A high continuous MetS score reflects a cascading synergy of lipotoxicity, glucotoxicity, and endothelial shear stress [20]. Unregulated central adiposity and dyslipidemia fuel a state of chronic, low-grade systemic inflammation [21], elevating circulating cytokines that permeate the blood–brain barrier and precipitate microglial activation [22]. Concurrently, peripheral insulin resistance disrupts central nervous system insulin signaling—a critical pathway for neuronal bioenergetics, synaptic plasticity, and the enzymatic clearance of amyloid-beta peptides [23]. Furthermore, the combined burden of hypertension and hyperglycemia drives cerebral microvascular remodeling and endothelial dysfunction [24, 25], leading to chronic hypoperfusion and the subcortical ischemic damage characteristic of vascular and mixed dementias [26, 27].

A pivotal clinical observation from our stratified analysis is the pronounced cognitive vulnerability among non-obese individuals (BMI < 24 kg/m^2^) harboring severe metabolic dysfunction. While possessing a “normal” outward phenotype, these individuals exhibited an 86% increased risk of dementia when their continuous MetS score was elevated. This directly implicates the “Metabolically Obese Normal Weight” (MONW) phenotype [28]. In MONW individuals, the primary site of fat storage shifts from subcutaneous depots to ectopic and visceral sites, resulting in profound, hidden insulin resistance [28, 29]. Our data compellingly suggest that visceral metabolic toxicity—captured accurately by the weighted components of the MetS score—is far more detrimental to the aging brain than total body mass [30].

This study has several strengths, including its prospective design, nationally representative sample, and use of a continuous, demographically standardized exposure metric. Integrating self-reports, medication history, and objective clinical measurements overcomes the inherent limitations of relying on a single ascertainment method. While self-reported diagnoses offer high specificity, they often suffer from low sensitivity due to underdiagnosis. Conversely, relying solely on clinical markers may miss individuals whose conditions are successfully managed pharmacologically. This composite approach minimizes misclassification bias by effectively capturing both undiagnosed and medically controlled cases. Several limitations should also be acknowledged. First, the observational design of the CHARLS cohort precludes causal inference. Second, the absence of neuroimaging data and genetic markers—notably APOE ε4 status—limits adjustment for key neurodegenerative pathways. Third, incident dementia was determined using a composite epidemiological algorithm; although validated for population screening, this approach lacks the specificity of biomarker‑based gold‑standard diagnosis. Fourth, metabolic severity was assessed only at baseline. Future studies incorporating longitudinal trajectories of metabolic scores are needed to clarify how the rate of metabolic deterioration affects cognitive outcomes. Fifth, our classification of lifestyle factors was constrained by data availability. We were unable to include physical activity—a critical determinant of both metabolic and cognitive health—due to high missing data rates (> 85%) in our analytical cohort. Furthermore, social activity was assessed based on participation during the single month prior to the interview, which may not capture long-term patterns of social engagement and could introduce exposure misclassification, potentially attenuating the observed associations. Sixth, we lacked granular pharmacological data. While specific medications such as metformin or certain antihypertensives may exert independent neuroprotective effects, the CHARLS dataset predominantly records broad medication categories, precluding our ability to adjust for specific drug classes.

## Conclusion

Quantifying metabolic severity with a continuous, age‑ and sex‑standardized score serves as a robust prognostic indicator for incident dementia, effectively capturing the dose–response nature of metabolic dysfunction. Our findings suggest that metabolic dysfunction acts along a destructive continuum, progressively eroding cognitive reserve. Integrating continuous metabolic scoring into routine geriatric assessments offers a pragmatic and highly scalable strategy for identifying subclinical neurocognitive risk—particularly in normal‑weight individuals who might otherwise remain undetected—thereby enabling earlier, targeted interventions to preserve brain health.

## Supplementary Information

Supplementary Material 1.

## Data Availability

The data supporting this study are publicly available from the CHARLS official website (http://charls.pku.edu.cn/).
