# Supplementary material for: Association between continuous metabolic syndrome score and incident dementia: a longitudinal study of middle-aged and older Chinese adults
Source: BMC Neurol. 2026 Jul 3;26:613. doi: 10.1186/s12883-026-05054-6 (PMC13625507; doi:10.1186/s12883-026-05054-6)
Supplement: Supplementary file 1 [file 12883_2026_5054_MOESM1_ESM.docx]

**Supplementary Method**

**The calculation of MetS score:**

For male aged<60 years: MetS score= -2.9092 + 0.0262*WC+ 0.3098*TG - 0.9440*HDL-C + 0.0097*MAP + 0.0745*FBG

For male aged ≥60 years: MetS score= -2.3741 + 0.0264*WC + 0.4933*TG - 0.9990*HDL-C + 0.0054*MAP + 0.0821*FBG

For female aged <60 years: MetS score= -2.4981 + 0.0199*WC + 0.5218*TG - 0.8616*HDL-C + 0.0110*MAP + 0.1074*FBG

For female aged ≥60 years: MetS score= -0.5682 + 0.0153*WC + 0.4587*TG - 1.3567*HDL-C + 0.0036*MAP + 0.0688*FBG

**Note:**

**WC**	Waist circumference (cm)

**TG**	Triglycerides (mmol/L)

**HDL-C**	High-density lipoprotein cholesterol (mmol/L)

**FBG**	Fasting blood glucose (mmol/L)

**SBP**	Systolic blood pressure (mmHg)

**DBP**	Diastolic blood pressure (mmHg)

**MAP**	Mean arterial pressure (mmHg) = DBP + (SBP - DBP) / 3

**Supplementary Table 1.** Odds ratios (ORs) and 95% confidence intervals (CIs) for the association between metabolic syndrome (MetS) score and subsequent dementia: multiple imputed.

| **MetS** **Score** | **No. of participants** | **No. of cases** | **Incidence rate (%)** | **OR (95% CI)** | **P** |
| --- | --- | --- | --- | --- | --- |
| Continuous | 4,920 | 667 | 13.56 | 1.12 (1.03, 1.23) | 0.011 |
| Categorical |  |  |  |  |  |
| Q1 (Lowest) | 1,266 | 139 | 10.98 | 1.00 (Reference) | - |
| Q2 | 1,167 | 153 | 13.11 | 1.19 (0.92, 1.54) | 0.180 |
| Q3 | 1,245 | 171 | 13.73 | 1.26 (0.98, 1.62) | 0.076 |
| Q4 (Highest) | 1,242 | 204 | 16.43 | 1.46 (1.12, 1.90) | 0.005 |
| P for trend |  |  |  |  | 0.005 |

Adjusted for age, sex, education, marital status, residence, body mass index, smoking, alcohol drinking, social activity, hypertension, diabetes, cardiovascular disease, and dyslipidemia.

**Supplementary Table 2.** Odds ratios (ORs) and 95% confidence intervals (CIs) for the association between metabolic syndrome (MetS) score and subsequent dementia: further adjusting for cognition score at baseline.

| **MetS** **Score** | **No. of participants** | **No. of cases** | **Incidence rate (%)** | **OR (95% CI)** | **P** |
| --- | --- | --- | --- | --- | --- |
| Continuous | 4,920 | 667 | 13.56 | 1.12 (1.01, 1.24) | 0.033 |
| Categorical |  |  |  |  |  |
| Q1 (Lowest) | 1,266 | 139 | 10.98 | 1.00 (Reference) | - |
| Q2 | 1,167 | 153 | 13.11 | 1.10 (0.82, 1.48) | 0.533 |
| Q3 | 1,245 | 171 | 13.73 | 1.19 (0.88, 1.61) | 0.253 |
| Q4 (Highest) | 1,242 | 204 | 16.43 | 1.50 (1.09, 2.07) | 0.014 |
| P for trend |  |  |  |  | 0.041 |

Adjusted for age, sex, education, marital status, residence, body mass index, smoking, alcohol drinking, social activity, hypertension, diabetes, cardiovascular disease, and dyslipidemia.
